# Relationship between tobacco smoking and hematological indices among Sudanese smokers

**DOI:** 10.1186/s41043-023-00493-0

**Published:** 2024-01-04

**Authors:** Izzut Awad Ahmed, Mahmood Abdalmonem Mohammed, Hussam Mohammed Hassan, Ibrahim Abdelrhim Ali

**Affiliations:** 1 Department of Physiology, Faculty of Medicine, The National Ribat University, Khartoum, Sudan; 2 Department of Physiology, Faculty of Medicine, Omdurman Islamic University, Khartoum, Sudan; 3 Department of Physiology, Faculty of Medicine, The Nile University, Khartoum, Sudan

**Keywords:** Tobacco, Cigarette smoking, Water pipes (shisha), CBC, Sudan

## Abstract

**Background:**

Tobacco Smoking is one of the leading causes of preventable morbidity and mortality in the world. It is well documented that tobacco smoking is risk factor for many diseases like: cancers, chronic respiratory and cardiovascular diseases, and the effects of tobacco smoking on hematological indices gets a little attention: the data is mostly inconsistent regarding the differential of WBCs, a conflicting studies described the effect of smoking on hemoglobin descriptive parameters and a regular monitoring of platelets count in smokers was advised.

**Objectives:**

The study aimed to evaluate the relationship between tobacco smoking and hematological parameters among Sudanese healthy Smokers at Bahri Town.

**Methods:**

This was a cross sectional study conducted during 2022 in Bahri town, Khartoum state. A total of 120 male subjects participated in this study. Of them, *60* healthy non-smokers participants (Control), and *60* age matched smokers who were smoking tobacco for a minimum of 1 year. Smokers group was divided into three major sub-groups with each group contains 20 subjects: Cigarettes smokers (CS), Water pipes (Shisha) smokers (WP) and both Cigarettes and water pipes (shisha) smokers (CSWP). Data was collected through questionnaire interviews and laboratory investigation. A sample of Five ml venous blood was taken for Complete blood count testing using Urite 3000 plus semi-automated hematology analyzer. Data were analyzed using SPSS version 25. Assocation between the variables were estimated and *p* value ≤ 0.05 was considered statistically significant.

**Results:**

Smokers had significantly higher RBCs count (*p* = 0.017), Hb level (*p* < 0.001), WBCs count (p = .017), Neutrophils (*p* < 0.001), MCH (*p* = 0.029), MCHC (*p* < 0.001), RDW (*p* < 0.001), and PDW (*p* < 0.001) compared to the non-smokers. In contrast, non-smokers had higher MPV (*p* < 0.001) and MCV (*p* < 0.001) levels than smokers. Between the non-smokers and different subtypes of the smokers (CS, WP & CSWP), there were significant differences between the subgroups for all hematological parameters except for PLTs and lymphocytes count. CS had lower levels of MCV (*p* < 0.001), MCHC (*p* < 0.001), HCT (*p* = 0.036), and RDW (*p* < 0.001) compared to the non-smokers, while both cigarette and shisha smokers had the higher levels of neutrophils count (*p* < 0.001) and PDW (*p* < 0.001) compared to the non-smokers.

**Conclusion:**

Smoking affects hematological parameters; smokers had significantly higher RBCs count, Hb level, WBCs count, Neutrophils, MCH, MCHC, RDW and PDW compared to the non-smoker group. WP smoking caused higher levels of RBCs, Hb, neutrophils, MCH and MCHC. PDW was high in smokers’ sub-groups compared to control group, while MPV was lower despite insignificant change In PLTs count.

## Introduction

Tobacco Smoking is one of the leading causes of preventable morbidity and mortality in the world [1]. It is well documented that tobacco smoking is risk factor for many diseases like: cancers, chronic respiratory and cardiovascular disease, but the association between smoking and hematological indices gets little attention. The ingredients in cigarette and water pipe (shisha) like nicotine leads to increase in leukocyte count [2, 3]. The Role of nicotine is suggested to be either by stimulation of hormones secretion that leads to an increase in leukocyte count [4], or due to the irritant effect of smoke on the respiratory system leads to inflammation and synthesis of cytokines which can influence the increase in leukocyte count [5]. Those results are thought to take their effects in a dose-dependent manner.

On the other hand, a relevant note during cessation of smoking shows associated decrease in leukocyte count [6]. The longer people abstain from cigarettes the lower the leukocyte count gets [7].

Regarding the differential of WBCs: Neutrophils, Basophils, Eosinophils, Lymphocytes and monocytes counts the data is mostly inconsistent.

Tobacco Smoking also affects red blood cells and hemoglobin. Hemoglobin and hematocrit are higher in smokers this Increase in hemoglobin concentration is believed to be mediated by exposure of carbon monoxide and some scientists suggested that an increase in hemoglobin level in the blood of smokers could be a compensatory mechanism [8, 9].

Red blood cells count (erythrocyte count) and Hematocrit (PCV) are associated with blood viscosity and clotting in smokers [10, 11]. Mean cell volume (MCV), Mean cell hemoglobin (MCH) and Mean cell hemoglobin concentration (MCHC) are three main red blood cell indices that help in measuring the average size and hemoglobin composition of the red blood cells.

A conflicting studies described the effect of smoking on the above parameters, greater values of MCV and MCH in smokers, in relation to non-smokers were confirmed by some studies [12, 13]. Some reports an increase in MCV and a decrease in MCH and MCHC levels in smokers [14].

Platelets is also an affected parameter Studies concluded that in smokers’ plasma fibrinogen concentration and platelet count increase significantly. And a regular monitoring of these two parameters in smokers was advised [15]. Platelet count—just like WBCs count—also shows a respectful decrease with smoking cessation [16].

This study aimed to investigate the effects of smoking on hematological indices, the additional data brought up by this study can help in raising awareness against smoking, and aid counselors with more information.

## Methods

### Study settings

An analytical case control community based study performed in Bahri Town, Khartoum state capital of Sudan in 2022 including *60* tobacco smoking male participants, between 18 and 60 years,  and *60* age-matched male smokers who are smoking tobacco for a minimum of 1 year. Twelve (12) social smoking centers (Café) were selected through clustered convenient non-probability sampling from the list of all legally registered centers in the locality of Bahri.

The smoking centers in which the study was conducted were selected randomly from 46 centers all over Khartoum north city which extracted randomly from the data base in Khartoum north locality,  and individuals within the centers was chosen by convenience none probability sampling.

The study population consists of 120 tobacco smoking centers visitors which divided into four subgroups: cigarettes smokers (CS) (*n* = 20), water pipe smokers (WP) (*n* = 20) and both cigarettes & water pipe smokers (CSWP) (*n* = 20) and control group of none smokers (NS) (*n* = 20). The sample size was calculated using the formula *N* = (1.962*p*q)/*d*2 where (*N*) refers to sample size, (*p*): expected prevalence of abnormal hematological indices among tobacco smokers, (*q*) 1−*p*, and (*d*): margin of error.

### Data collection and analysis

Questionnaire interviews with all participants were done covering information about age, occupation, marital status, educational level, level of income, age at first started smoking, duration of smoking, and the average cigarettes, and water pipes (shisha) per month.

Measurement of height (cm) and weight (Kg) were done using standard techniques.

Body mass index (BMI) was calculated by dividing the weight (Kg) by the square meter of the height. Arterial blood pressure (ABP) was measured using mercury sphygmomanometer.

Five ml of venous blood was drawn from the brachial veins, collected by the standard procedure from each participant under complete aseptic conditions and stored in containers contain EDTA (ethylene diamine tetra acetate, an anticoagulant) to prevent it from clotting.

Complete blood count was done using *Urite 3000 plus* semi- automated hematology analyzer.

All the data collected in this study were analyzed using Statistical Package for Social Sciences (SPSS) version 25. In comparing the results between the control and smoker’s groups, The Mann Whitney U test and Independent *T*- test were used for the analyzes and a *p* value of ≤ *0.05* was considered statistically significant.

### Ethical consideration

Informed written consent was signed by all participants after fully explaining the objectives of the project. Also, Approval of the ethical committee was taken from the Faculty of Medicine, The National Ribat University, and Khartoum North Locality Institute before study initiation.

## Results

A total of 120 male participants were included in the study. Participants’ ages ranged from 18 to 60 years, with a median age of 24 for both smokers and non-smokers. There were no substantial differences between the groups in terms of median systolic and diastolic blood pressure, although the smoker group had a slightly higher median BMI (21.39) than the non-smoker group (20.75) (Table 1).

**Table 1:** Baseline characters of the study population.

Variable	Smokers(*n* = 60)	Nonsmokers (*n* = 60)	*p* value
Age (years)	24 (18–45)	24 (20–60)	0.736
BMI (kg/m^2^)	21.39 (14.17–32.14)	20.75 (12.5–37.7)	0.399
Systolic blood pressure (mmHg)	120 (90–165)	120 (100–165)	0.651
Diastolic blood pressure (mmHg)	80 (60–110)	80 (60–100)	0.585

Data expressed as medians (range)

Regarding the hematological parameters of the participants, the results showed that participants from the smokers group had significantly higher RBCs count (*p* = 0.017), Hb level (*p* < 0.001), WBCs count (*p* = 0.017), Neutrophils (*p* < 0.001), MCH (*p* = 0.029), MCHC (*p* < 0.001), RDW (*p* < 0.001), and PDW (*p* < 0.001) compared to the non-smoker group. In contrast, non-smokers had higher MPV (*p* < 0.001) and MCV (*p* < 0.001) levels than smokers (Table 2).

**Table 2:** Comparison of hematological parameters between smokers and non-smokers. Data are presented as means ± SD or medians (range)

Hematological parameters	Nonsmokers( n = 60)	Smokers ( n = 60)	*p* value
RBCs (× 10^12^/L)	5.18 ± 0.47	5.37 ± 0.42	0.017*
Hb (g/dl)	15.17 ± 1.23	16.17 ± 1.58	0.000*
HCT (%)	46.1 (37.50–60.50)	45.6 (36.3–52.9)	0.122
WBCs (× 10^9^/L)	4.75 (2.4–8.0)	5.2 (3.2–11.1)	0.023*
Neutrophils (× 10^9^/L)	1.7 (0.4–12.50)	2.6(2.0–3.88)	0.000*
Lymphocytes (× 10^9^/L)	2.0 (1.1–4.8)	1.9 (1.1–7.4)	0.118
Mixed	0.5 (0.1–1.8)	0.6 (0.2–2.0)	0.509
MCV (fl)	90.1 (69.8–117.5)	85.8 (67.1–95.8)	0.000*
MCH (Pg)	29.65 (21.5–35.7)	30.55 (21.2–34.0)	0.029*
MCHC (g/dl)	32.45 (26.6 -36.9)	35.5 (31.6–37.20)	0.000*
PLT (× 10^9/L)	258 (126.0–425.0)	256 (104.0–443.0)	0.844
RDW (%)	14.1 (11.4–16.8)	15.75 (13.6–20.5)	0.000*
MPV (fl)	15.05 (9.3–20.3)	6.65 (5.1–9.30)	0.000*
PDW (fl)	9.5 (8.1–15.2)	14.9 (12.5–18.1)	0.000*

*Significance of difference in Mann–Whitney test for data following non-normal distributed and *t*-test for normal distributed data

**Fig. 1:**
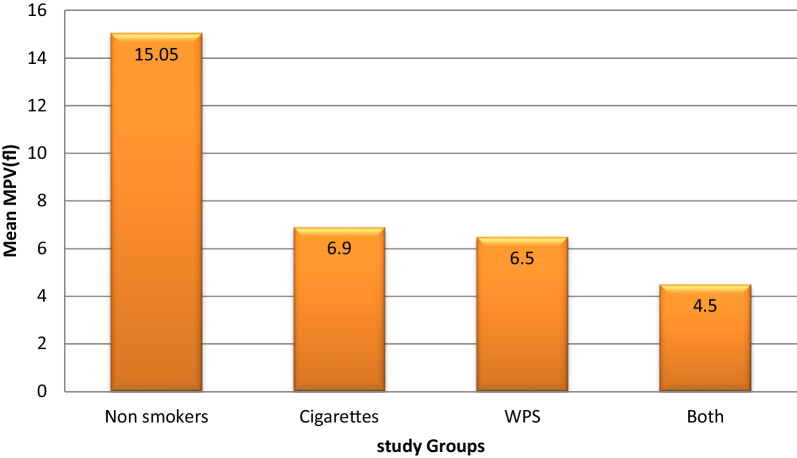
Difference in mean MPV between non-smokers and smokers groups

**Fig. 2:**
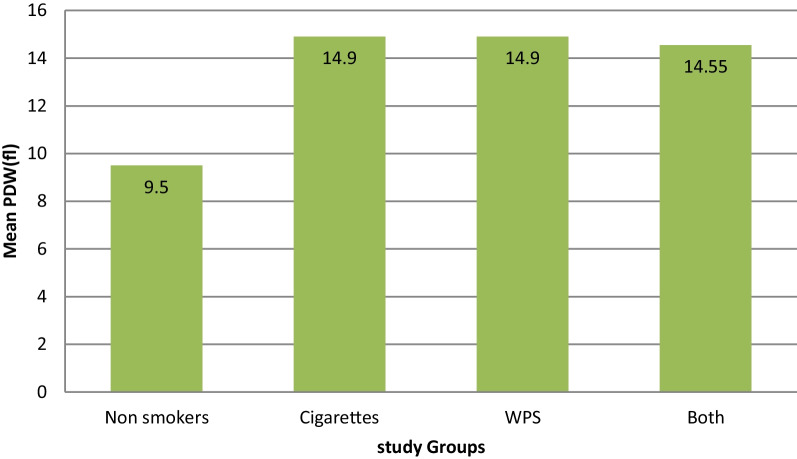
Difference in mean PDW between non-smokers and smokers groups

Furthermore, we examined the pattern of differences in hematological parameters between the non-smoker group and different subtypes of the smokers group: Cigarettes smokers (CS) group, water pipes (Shisha) (WP) smokers group and both Cigarettes and water pipes (CSWP) smokers group. There were significant differences between these subgroups for all of the assessed hematological parameters except for PLTs and lymphocyte count. The results showed cigarette smokers had lower levels of MCV (*p* < 0.001), MCHC (*p* < 0.001), HCT (*p* = 0.036), and RDW (*p* < 0.001) among smokers compared to the non-smokers, while both cigarette and shisha smokers had the higher levels of neutrophils count (*p* < 0.001) and PDW (*p* < 0.001) among smokers compared to the non-smokers (Table 3), (Fig. 1–2).

**Table 3:** Comparison of hematological parameters between non- smokers and Smokers sub-groups

Hematological parameters	Nonsmokers (*n* = 60)	Cigarettes smokers (*n* = 20)	Shisha smokers (*n* = 20)	Both smokers (*n* = 20)	P1	P2	P3
RBCs (× 10^12^/L)	5.18 ± 0.47	5.25 ± 0.29	5.52 ± 0.52	5.35 ± 0.42	0.613	0.007*	0.142
Hb (g/dl)	15.17 ± 1.23	15.6 ± 1.28	16.84 ± 1.6	16.06 ± 1.63	0.089	0.001*	0.011*
HCT (%)	46.1 (37.5–60.5)	44.65 (36.3–48.5)	47.75 (38.4–52.9)	45.95 (36.3–50.4)	0.036*	0.898	0.194
WBCs (× 10^9^/L)	4.75 (2.4–8.0)	5.75 (3.3–9.9)	4.9 (3.2–11.1)	5.1 (4.0–7.7)	0.023*	0.178	0.236
Neutrophils (× 10^9^/L)	1.7 (0.4–12.50)	2.65 (1.3–6.1)	2.7 (1.1–7.4)	2.4 (1.3–5.1)	0.001*	0.001*	0.004*
Lymphocytes (× 10^9^/L)	2.0 (1.1–4.8)	2.0 (1.3–6.1)	1.8 (0.3–3.8)	1.95 (1.1–4.0)	0.705	0.066	0.273
Mixed	0.5 (0.1–1.8)	0.6 (0.2–1.5)	0.65 (0.2–2.0)	0.55 (0.2–1.6)	0.474	0.310	0.737
MCV (Fl)	90.1 (69.8–117.5)	84.5 (67.1–91.0)	87 (74.3–95.0)	85.1 (77.6–93.6)	0.001*	0.013*	0.001*
MCH (Pg)	29.65 (21.5–35.7)	30.15 (21.2–32.1)	30.95 (25.5–34.0)	30.2 (26.6–33.7)	0.359*	0.010*	0.248
MCHC (g/dl)	32.45 (26.6–36.9)	32.25 (31.6–36.7)	35.6 (34.4–37.2)	35.5 (33.6–37.0)	0.001*	0.001*	0.001*
PLT (× 10^9^/L)	258 (126–425)	267.5 (104–354)	249.5 (143–443)	266 (106–359)	0.494	0.434	0.609
RDW (%)	14.1 (11.4–16.8)	15.15 (13.6–19.9)	15.95 (14–18.1)	16.0 (13.9–20.5)	0.001*	0.001*	0.001*
MPV (fl)	15.05 (9.3–20.3)	6.9 (5.7–8.6)	6.5 (5.8–9.3)	6.8 (5.1–8.3)	0.001*	0.001*	0.001*
PDW (fl)	9.5 (8.1–15.2)	14.9 (13.6–17.9)	14.9 (13.6–17.9)	14.55 (12.5–18.1)	0.001*	0.001*	0.001*

Data are presented as means ± SD or medians (range)

*Significance of difference in Mann–Whitney test for data following non-normal distributed and *t*-test for normal distributed data

*P1*: non-smokers versus Cigarettes Smokers, *P2*: non-smokers versus Shisha Smokers, *P3*: non-smokers versus Both (Cigarettes and Shisha) smokers

## Discussion

Smoking is one the biggest health threats today, and while many of its effects are well documented a handful are still in debate such as its relationship to hematological indices. Hence this study was necessary to add to the present data. In general, the results indicated no significant difference between smokers and non-smokers in age, weight, and blood pressure values, but significant differences in hematological parameters were noted, and an increase in hemoglobin and RBCs was noticed. The data also suggested an increase in WBC count and neutrophils count among smokers compared to non-smokers. However, in PLT counts the analysis demonstrated no statistical difference.

To put these results in context, we found a significant increase in Hb and RBCs just like many studies did, for instance, Nadia et al. and Rawia Osman Ali studies [17, 18] which were conducted in Sudan, a study even detailed that water pipes smokers especially demonstrated a significantly higher Hb value [19], which was in agreement with our findings. Another study [20] found that both Hb and RBC counts were significantly higher in WP smokers than in cigarette smokers.

Our results were generally consistent with the literature, the only contradiction was that our study found the HCT values to be significantly lower in cigarette smokers and no significant increase in WP smokers or both cigarette and WP smokers when compared to non-smokers, while most of those studies found a significant increase in HCT levels in smokers compared to non-smokers [17–22], some even demonstrated a higher increase in HCT levels among WP smokers compared to cigarette smokers [19]. More studies make it evident that there is some agreement on the effects of smoking on Hb and our data is building on this existing evidence [9, 21, 23, 24].

Suggested explanations for these findings can be that carbon monoxide released from smoke combines with hemoglobin to form carboxyhemoglobin that causes tissue hypoxia which leads to an increase in erythropoietin secretion and erythropoiesis. Carbon monoxide also increases capillary permeability and decreases plasma volume which mimics relative polycythemia [25].

Comparing the findings of MCV, MCH and MCHC to the present literature is not as straightforward, as the literature is very inconsistent, many studies [9, 17, 18, 21–23] found an increase in MCV which in direct contradiction with our findings, we found MCV to be significantly lower in all smokers group compared to non-smokers. One study [20] did not find any significant increase in MCV. Regarding the MCH our results showed an increase in the smokers' group but only significant in WP smokers, a similar study found an increase in MCH but more in cigarette smokers than WP smokers [20], and many studies found this increase in MCH [9, 17, 18] except one. The MCHC findings provided by our study were significantly higher in WP smokers and Both smokers and lower in cigarette smokers, a consistent result was found by a study [20] suggesting MCHC was significantly higher among smokers but more in WP smokers than cigarette smokers, some studies found MCHC to be significantly increased [17], while some found it to be lower in smokers than non-smokers [21], while some found no significant difference.

WBC count was higher in all smoker's groups and significantly in cigarette smokers, congruent with many studies in the literature [9, 19, 22–24], only one study went against this consensus and found a significant decrease in total WBC count. We found a significant increase in neutrophil count and no significant increase in lymphocyte count, this was an area for debate in the literature, and some were in line with our results [10, 18]. Some showed an increase in both neutrophils and lymphocytes [22, 23], one study found no significance in both neutrophils and lymphocytes [17], and one study found contradictory results that showed a significant decrease in neutrophil count and a significant increase in lymphocyte count. These findings might be due to the irritant effect of the chemicals found in cigarettes, the major one being nicotine. The role of nicotine is suggested to stimulate hormone secretion that leads to an increase in leukocyte count [4], also the irritant effect of smoke on the respiratory tree leads to inflammation and synthesis of cytokines which can influence the leukocyte count [5]. Those results are thought to take effect in a dose-dependent manner. Furthermore, on a relevant note cessation of smoking is associated with a decrease in leukocyte count [6].

Our study found that PLT count was insignificantly higher among cigarette smokers and insignificantly lower among water pipe smokers. One study found a significant increase in PLT count [21] in water pipe smokers, while another found a significant decrease [18], but two studies found no significant increase or decrease [17, 22]. It was reported that the hormonal pathways regulating platelet production may be potentially impaired following smoking inducing the production of platelets and increased platelet count. But when it’s chronic had higher circulating thrombopoietin levels (a humoral growth factor that primes platelet activation and production) than nonsmoking controls [26].

PLT indices and RDW showed significant results, RDW and PDW were both significantly higher in smoker groups, and MPV showed significantly lower results in smoker groups, these findings seem to be different than what is reported by other literature, many studies found no significant effect on MPV [2, 22, 27] and no significant changes in PDW [21, 22] and RDW [9]. Only a few studies found significant differences in platelet indices, one found a significantly lower MPV in smokers’ groups [21] which disagreed with our study, and another found the PDW results to be higher in smoker groups [27] both of which agree with our findings.

The use of the above result we recommend research projects on cessation period to develop and tailor new Sudanese smoking cessation treatments, including physical and mental treatment modalities using psychometric assessments according to smoking status and physical modalities of investigations.

Tailoring treatment based on the results of these physical and psychological assessments and interventions may increase the success rate of smoking cessation among the Sudanese population.

### Limitations of the study

This study had several limitations. Firstly, the study sample was small, which limits our ability to generalize these results. This study did not include female smokers despite the observed recent increase in the smoking rate among females, especially shisha. Finally, this study could not estimate the effects of passive smoking on hematological indices.

## Conclusions

The results indicate that smoking affects hematological parameters, smokers had significantly higher RBC count, Hb level, WBC count, Neutrophils, MCH, and MCHC compared to the non-smoker group. This generally supports the existing literature. 

## Data Availability

The data generated in this study are available from the corresponding author upon reasonable request with a completed Materials Transfer Agreement, Excluding the materials including personally identifiable information.
